# The Effects of Interval Resistance—Aerobic Training and Fisetin Supplementation on Asprosin and Selected Adipokines in Obese Men: A Double-Blind Randomized Control Trial

**DOI:** 10.3390/nu18030433

**Published:** 2026-01-28

**Authors:** Mehran Alipour, Ayoub Saeidi, Keyvan Hejazi, Rashmi Supriya, Hassane Zouhal

**Affiliations:** 1 Department of Physical Education and Sport Sciences, Faculty of Humanities and Social Sciences, University of Kurdistan, Sanandaj 6617715175, Kurdistan, Iran; alipour.mehran1212@gmail.com; 2 Department of Sport Sciences, Faculty of Sport Sciences, Hakim Sabzevari University, Sabzevar 9617976487, Razavi Khorasan, Iran; 3 Department of Sports and Health Sciences, Faculty of Arts and Social Sciences, Academy of Wellness and Human Development, Hong Kong Baptist University, Kowloon Tong, Hong Kong SAR 999077, China; 4 Centre for Health and Exercise Science Research, Hong Kong Baptist University, Kowloon Tong, Hong Kong SAR 999077, China; 5 International Institute of Sport Sciences (2I2S), 35000 Rennes, France

**Keywords:** interval resistance training, fisetin, asprosin, obesity

## Abstract

**Objective:** This double-blind, parallel-group randomized controlled trial is the first to investigate the synergistic effects of interval resistance plus progressive aerobic training with fisetin supplementation on adipokines in obesity. **Methods:** Sixty sedentary men with obesity (BMI < 30 kg/m^2^) completed 12 weeks of thrice-weekly interval resistance training (eight exercises, 3 × 13 reps at 60% 1RM with 20% 1RM active rest), immediately followed by staged aerobic bouts (50–70% HRmax). Participants were randomized into the control-placebo (P), fisetin (F; 200 mg/day), training-placebo (TP), or training + fisetin (TF) groups. The primary outcomes were asprosin, MCP-1, and adiponectin; secondary outcomes included leptin and lipid profile. Data were analyzed via ANCOVA with Bonferroni post hoc tests. **Results:** Statistical analyses were conducted following the intention-to-treat (ITT) principle using an analysis of covariance (ANCOVA) model, which revealed extensive effects of the interventions on the participants’ anthropometric and biochemical indices. Regarding body composition, after adjusting for baseline values, a significant difference in mean body weight was observed between groups (F (3, 55) = 9.444, *p* < 0.001, ηp^2^ = 0.340); Bonferroni post hoc tests confirmed that the training plus fisetin (TF), training-placebo (TP), and fisetin (F) groups all achieved significant weight loss compared to the placebo (P) group. Furthermore, body mass index (BMI) showed a significant inter-group difference (*p* = 0.021), with post hoc analysis revealing that only the TF group reached a statistically significant reduction compared to the placebo (*p* = 0.024; 95% CI [−3.760, −0.172]). In the assessment of biochemical and inflammatory variables, the interventions exerted a highly significant effect on asprosin (F (3, 55) = 36.047, *p* < 0.001; ηp^2^ = 0.663) and MCP-1 (F (3, 55) = 29.570, *p* < 0.001; ηp^2^ = 0.617). The findings indicated that the TF group experienced the most substantial reductions in both asprosin (−60.71%) and MCP-1 (−46.50%) levels. Regarding adipokines, significant increases in adiponectin levels were observed in the TP (29.38%) and TF (27.67%) groups (*p* < 0.05), whereas changes in leptin were statistically significant only in the TF group relative to the placebo (*p* = 0.049). The lipid profile results indicated a statistically significant difference in the TF group in improving all markers; this group achieved greater reduction compared to other groups, including reductions in LDL-C, triglycerides (TG), and total cholesterol (TC) (*p* < 0.001), while simultaneously showing a significant elevation in HDL-C. Post hoc analyses confirmed robust statistical differences in all lipid parameters for both the TF and TP groups compared to the placebo group (*p* < 0.05), whereas the placebo group experienced a deterioration in status characterized by a significant increase in LDL-C (*p* = 0.027) and a significant decline in HDL-C concentrations (*p* = 0.006). **Conclusions**: In conclusion, 12 weeks of combined interval resistance–aerobic training and fisetin supplementation significantly reduced pro-inflammatory adipokines and improved lipid profiles in obese men. These findings suggest that asprosin serves as a potential modulator in metabolic risk reduction; however, since direct mechanistic assays were not conducted, these implications remain hypothetical. Future research employing molecular readouts is warranted to confirm the underlying pathways involved.

## 1. Introduction

Obesity, recognized as a paramount public health challenge of the 21st century, is intricately linked to the pathogenesis of metabolic dysregulation, cardiovascular diseases, and chronic systemic inflammation [1]. Expanding beyond its traditional role as a mere energy reservoir, adipose tissue functions as a sophisticated endocrine organ that modulates inflammatory cascades and insulin sensitivity through the secretion of diverse adipokines, including leptin, adiponectin, asprosin, and monocyte chemoattractant protein-1 (MCP-1) [2,3]. In the obese state, the homeostatic balance of these signaling proteins is profoundly disrupted—characterized by elevated leptin and MCP-1 concentrations alongside diminished adiponectin levels—thereby establishing a pro-diabetogenic environment that facilitates the progression of insulin resistance and type 2 diabetes mellitus [4,5,6]. Recent physiological evidence suggests that exercise interventions, particularly interval resistance training (IRT), may offer superior efficacy compared to conventional modalities in ameliorating these biomarkers, primarily due to the induction of heightened metabolic stress and a more pronounced excess post-exercise oxygen consumption (EPOC) [7,8,9].

Asprosin, a novel white adipose tissue-derived glucogenic hormone, exerts a direct regulatory influence on glucose homeostasis; clinical investigations have demonstrated a robust positive correlation between serum asprosin levels and body mass index (BMI), adiposity, and insulin resistance in obese cohorts [10,11]. Chronic pathological elevations of this hormone trigger excessive hepatic glucose production and induce metabolic stress within pancreatic beta cells, further exacerbating glycemic instability [12]. Given its pivotal role in glucose dysmetabolism, asprosin has emerged as a primary outcome measure for evaluating the efficacy of metabolic interventions, and modulating it signaling pathways represents a promising therapeutic target for enhancing insulin sensitivity in obesity management.

Complementing physical activity, natural flavonoids such as fisetin have demonstrated significant potential in optimizing adipokine profiles, attributed to their potent anti-inflammatory and antioxidant properties [13,14,15]. Despite the existing body of evidence, the potential synergistic effects of combining IRT and aerobic exercise with fisetin supplementation on adipokine dynamics remain unexplored in the current literature. This study is predicated on the primary hypothesis that a multi-modal intervention—integrating IRT, aerobic training, and fisetin consumption—will result in a more substantial reduction in asprosin and MCP-1 levels, coupled with a significant elevation in adiponectin, compared to independent interventions. Furthermore, we hypothesize that, with this combinatorial approach, we can expect certain body composition indices and insulin sensitivity conditions among obese males. Consequently, the present research aims to investigate the impact of a 12-week regimen of combined interval resistance and aerobic training, supplemented with fisetin, on circulating asprosin and related adipokines in this population.

## 2. Materials and Methods

### 2.1. Participants and Ethics Approval

This randomized controlled trial adopted a parallel-group design and initially recruited 107 adult males with obesity who volunteered to participate. Following eligibility screening, 60 individuals meeting all inclusion criteria were enrolled in the study.

Eligible participants were sedentary men with obesity (BMI > 30 kg/m^2^) who reported no structured physical activity and had abstained from alcohol consumption for at least six months prior to the study. To ensure baseline health status, inclusion required a resting blood pressure below 140/90 mmHg and fasting blood glucose not exceeding 126 mg/dL; concurrently, smokers and individuals utilizing antihypertensive, statin, or glucose-lowering medications were excluded from the enrollment process. Exclusion criteria encompassed any physical limitations or joint disorders, endocrine, metabolic, or cardiovascular disease, as well as the use of prescription medications or nutritional supplements known to influence muscle or adipose tissue metabolism. Additionally, individuals consuming over-the-counter products containing caffeine, protein, or similar bioactive compounds were excluded.

A familiarization session was conducted one week prior to initiating the intervention protocols, during which the study design and procedures were thoroughly explained. All participants signed a written informed consent form and completed the Physical Activity Readiness Questionnaire (PAR-Q) a validated and reliable screening tool widely applied across diverse populations to assess readiness for exercise participation. Ethical approval for all study procedures was obtained from the Research and Ethics Committee of the Hakim Sabzevari University, (Ethics code: IR.HSU.REC.1404.044). The trial was prospectively registered at the Iranian Registry of Clinical Trials (https://irct.behdasht.gov.ir/trial/87431 (accessed on 25 October 2025) under registration number (IRCT20120129008863N14). All processes adhered to the principles outlined in the latest revision of the Declaration of Helsinki [16].

Physical assessments were performed by a qualified examiner during the initial visit. Baseline evaluations including anthropometric measurements, body composition analysis, and biochemical assays were subsequently conducted during a second visit. Assessments were performed at two time points: baseline and after completion of the 12-week training program. Post-intervention testing was scheduled 48 h after the final exercise session for all groups, while baseline testing occurred 48 h before initiating training and/or supplementation. All measurements were undertaken under standardized environmental conditions, in the morning, and within a one-hour time frame to minimize diurnal variation.

**Figure 1:**
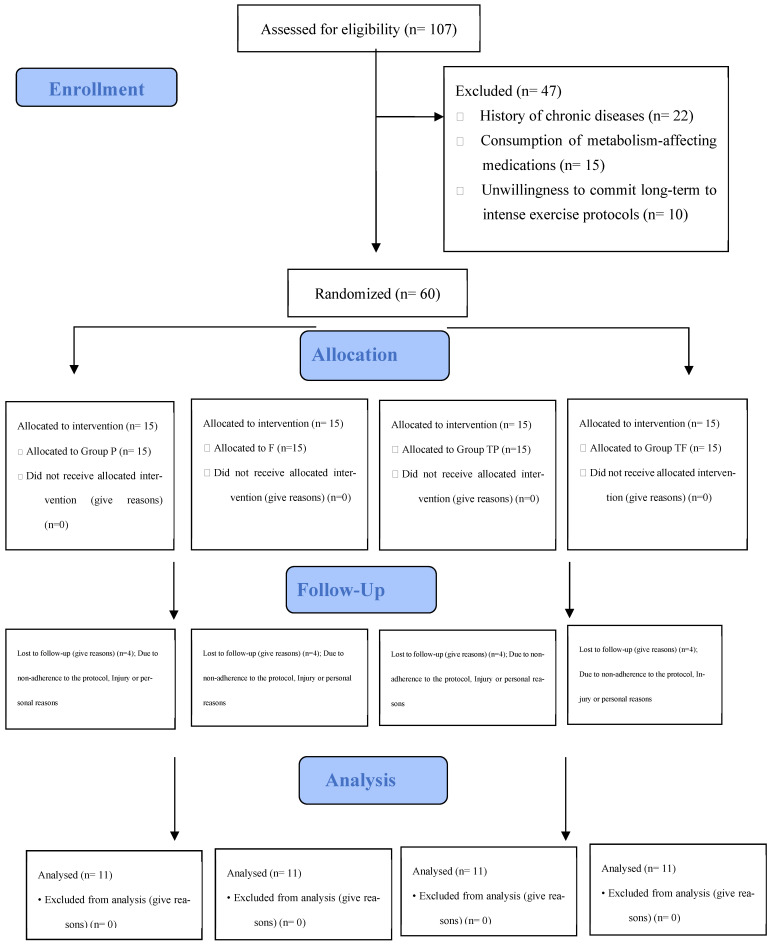
The details of the participant registration, screening, and random assignment process.

Randomization was implemented using a computer-generated block randomization list (block size = 4), ensuring equal allocation into four intervention arms: Control-Placebo (P), fisetin (F) (200 mg/day), Training-Placebo (TP), or Training-Fisetin (TF) (Figure 1). Allocation concealment was achieved through the use of opaque, sealed envelopes prepared by an independent researcher not involved in participant recruitment or data collection. This study followed a double-blind design for the supplementation aspect, meaning participants were unaware of whether they received placebo or fisetin. The coaches or trainers were unaware of the assignment/task of the supplementary groups.

To monitor and partially standardize dietary intake, participants adhered to a standardized diet for 72 h prior to both baseline and final measurements. The final randomized sample included 60 participants (n = 15 per group). All attrition events and their underlying reasons were systematically documented, and participant flow is visually represented in the CONSORT diagram to ensure methodological transparency (Figure 1).

### 2.2. Training Protocol

Participants’ one-repetition maximum (1RM) was estimated using the Brzycki equation after a standardized warm-up. Following light preparatory sets, each participant selected a load they could perform for up to 10 repetitions. If more than 10 repetitions were achieved, load was increased after a brief rest and the attempt repeated until a load producing ≤10 repetitions was identified. The final load and repetition count were recorded and used to calculate 1RM as:

1RM (kg) = weight (kg)/1.0278 − (0.0278 × repetitions).

The resistance program was implemented as an Interval, alternating lower- and upper-body exercises. Eight exercises were included in each session in the following sequence: back squat, bench press, leg curl (machine), biceps curl, leg press, barbell shoulder press, leg extension (machine), and lat pulldown. Sessions were supervised by certified exercise professionals to ensure correct technique and participant safety.

The exercise intervention was implemented based on a progressive model, where each training session comprised eight distinct exercises performed in three sets. Each set consisted of 13 repetitions at an intensity of 60% 1RM, immediately followed by an active recovery period involving 15 repetitions at 20% of the 1RM. To adhere to the principle of progressive overload, each participant’s 1RM was reassessed every four weeks, allowing for the recalibration of the 60% and 20% training loads for the subsequent mesocycle. Furthermore, to ensure optimal recovery and achieve the recorded mean adherence rate of 85% across all groups, the program structure incorporated 90 s rest intervals between sets and 2 min rest periods between different exercises [17].

Immediately following the interval-resistance circuit, participants performed a treadmill aerobic bout with progressive intensity and duration across the 12-week intervention: weeks 1–4: 15 min at 50% HRmax; weeks 5–8: 20 min at 60% HRmax; and weeks 9–12: 25 min at 70% HRmax. Maximum heart rate (HRmax) was estimated using the age-predicted equation (HRmax = 220 − age). Aerobic exercise intensity was prescribed relative to the estimated HRmax and continuously monitored in real time using Polar chest-strap heart-rate monitors; heart-rate data were recorded and workloads were adjusted as needed to maintain the target intensities.

Each session began with a 10 min standardized warm-up and concluded with a 10 min cool-down. Adherence, session attendance, and any adverse events were documented throughout the study. Adherence to the intervention was high, with an average session attendance of 92%. Supplement compliance was monitored via bi-weekly pill counts, showing a 96% adherence rate. Habitual dietary intake remained stable throughout the 12 weeks, as evidenced by periodic 3-day food records showing no significant inter-group differences in total energy or macronutrient intake. No adverse clinical events were associated with either the exercise protocol or fisetin supplementation. All sessions were conducted under direct supervision to maximize safety and protocol fidelity.

Control participants were instructed to maintain their usual daily activities and to refrain from initiating any new structured exercise programs during the 12-week period. All participants provided informed consent and were free to withdraw at any time. The study procedures conformed to relevant ethical guidelines, and any deviations or adverse events were recorded and reported.

### 2.3. Supplementation Protocol

In this study, Fisetin supplementation (200 mg/day; Novusetin™, Orleans, NY, USA) or a placebo was administered in a double-blind manner, ensuring that both the participants and the researchers responsible for outcome assessment and statistical analysis remained masked to the group assignments. Participants in the supplement group consumed one 200 mg capsule daily immediately after breakfast [18], while the control group received starch-based placebo capsules that were identical in size, shape, and color to the active supplement to maintain rigorous blinding. Adherence to the supplementation protocol was monitored through bi-weekly pill counts of the returned containers; notably, no serious adverse events were reported throughout the intervention period.

To minimize potential dietary confounders, all participants completed a three-day dietary recall at two critical time points: during the three days preceding the baseline blood sampling, and again in the three days prior to post-intervention blood collection. This approach facilitated the monitoring of dietary patterns and ensured that variations in energy or nutrient intake did not influence the biochemical outcomes under investigation.

### 2.4. Dietary Monitoring and Analysis

To monitor and partially standardize dietary intake without imposing a strict metabolic diet, participants were instructed to maintain their habitual nutritional patterns throughout the 12-week study period. In this regard, all subjects recorded their dietary intake at specific intervals, including at baseline and post-intervention, using a three-day food record (comprising two weekdays and one weekend day) to verify the stability of their eating habits and ensure that no significant dietary shifts occurred during the intervention. Furthermore, participants received bi-weekly verbal reminders to adhere to their baseline eating patterns, and continuous communication with the trainers served as an additional measure to prevent spontaneous dietary changes during the intervention.

These records were analyzed with Diet Analysis Plus v.10 software (Cengage Learning, Boston, MA, USA), which facilitated estimation of daily total caloric intake alongside macronutrient distribution, including carbohydrate, fat, and protein consumption (Table 1). To balance protocol feasibility with participant burden, dietary intake was monitored at baseline and week 12. Participants were frequently reminded during exercise sessions to maintain their baseline eating habits to ensure that the observed metabolic changes were primarily attributable to the intervention rather than spontaneous dietary shifts.

### 2.5. Blood Biomarkers

To ensure a rigorous and focused analysis, study outcomes were prospectively categorized into primary and secondary endpoints. Primary outcomes included the changes in circulating levels of asprosin, MCP-1, and adiponectin, as these were the central variables for testing our main hypothesis. Secondary outcomes comprised changes in anthropometric indices (body weight and BMI) and the lipid profile (TG, TC, LDL-C, and HDL-C). All statistical interpretations and multiple comparison adjustments were prioritized according to this hierarchy.

Venous blood samples were collected from the right antecubital vein following a 12 h overnight fast at two time points: 48 h before the first training session and 48 h after the last session; all samplings were performed between 8:00 and 10:00 AM to control for circadian variation, and each sample was collected in EDTA-coated Vacutainer tubes, centrifuged at 3000 rpm for 10 min, and stored at −70 °C until biochemical assays were performed. Serum concentrations were analyzed using specific human enzyme-linked immunosorbent assay (ELISA) kits, among which Asprosin, MCP-1, and adiponectin were designated as primary outcomes; specifically, Asprosin concentration was measured with a sandwich ELISA kit (Elabscience Biotechnology, Wuhan, China; Catalog No. E-EL-H0515; detection range 0.156–10 ng/mL; sensitivity 0.094 ng/mL; Intra-assay CV < 10% and Inter-assay CV < 10%), MCP-1 concentration with a sandwich ELISA kit (R&D Systems, Minneapolis, MN, USA; Catalog No. DCP00; sensitivity 10 pg/mL; Intra-CV = 7.8% and Inter-CV = 6.7%), and adiponectin concentration with an ELISA kit (Biovendor, Brno, Czech Republic; Catalog No. RD195023100; sensitivity 26 ng/mL; Intra-CV = 4.9% and Inter-CV = 6.7%). Furthermore, the lipid profile, anthropometric indices, and other markers were considered as secondary variables to adopt a structured approach in addressing multiplicity; in this regard, total cholesterol (TC) and triglycerides (TGs) were measured using enzymatic methods (CHOD-PAP), while HDL-C and LDL-C were measured via photometric methods (quantitative diagnostic kit by Pars Azmoon, Tehran, Iran; with a coefficient of 1.8% and sensitivity of 1 mg/dL for HDL, and a coefficient of 1.2% and sensitivity of 1 mg/dL for LDL), and leptin concentration was also determined with an ELISA kit (Biovendor, Czech Republic; Catalog No. RD191001100; sensitivity 0.2 ng/mL; Intra-CV = 5.9% and Inter-CV = 5.6%).

In interpreting asprosin levels, it is crucial to consider the methodological variability and existing challenges in the assay of this hormone. Although specific ELISA kits (such as Elabscience) were employed, differences in kit sensitivity and laboratory procedures can lead to fluctuations in results reported across various studies, further emphasizing the necessity for standardizing measurement protocols in future research.

### 2.6. Statistical Analysis

Statistical analyses were performed using IBM SPSS Statistics (version 26). To mitigate potential attrition bias and preserve the integrity of the initial randomization, all analyses followed the ITT principle, accounting for all 60 randomized participants. Missing data points resulting from the 16 withdrawals were managed using group-based mean imputation. The normality of data distribution and homogeneity of variances were rigorously verified via Shapiro–Wilk and Levene’s tests, respectively. Initially, a one-way ANOVA was executed to ensure the absence of significant baseline disparities among the four experimental cohorts. To precisely evaluate post-intervention differences while neutralizing the confounding influence of initial values, a one-way Analysis of Covariance (ANCOVA) was employed, utilizing baseline measures as the covariate. Furthermore, a 4 × 2 Repeated Measures ANOVA examined the temporal dynamics of dependent variables. In alignment with a conservative statistical interpretation, the Group × Time interaction was prioritized as the primary evidence of efficacy; within-group changes were treated as secondary and interpreted with caution in the absence of a significant interaction. A Per-Protocol (PP) analysis (n = 44) was also conducted as a sensitivity check to delineate the distinction between pragmatic effectiveness ITT and maximal potential efficacy (PP). Upon detection of a significant main effect or interaction, the Bonferroni post hoc test was utilized for specific pairwise comparisons. The magnitude of intervention effects was quantified using partial eta squared (ηp^2^), with benchmarks set at small (0.01), medium (0.06), and large (≥0.14). To ensure robustness beyond dichotomous *p*-values, primary findings are reported alongside their 95% Confidence Intervals (95% CI). Finally, the interrelationships between the magnitude of change (Delta) in significantly altered variables, including asprosin and metabolic markers, were assessed using Pearson’s correlation coefficient under the ITT framework (N = 60). The significance threshold for all analyses was set at *p* < 0.05.

## 3. Results

Details regarding the enrollment process, randomized allocation, and participant flow are presented in Figure 1 (CONSORT Diagram); additionally, the specific reasons for subject attrition in each group have been documented separately in Table 2.

The characteristics of the participants in the experimental and placebo groups are shown in Table 3. Participants’ age, height, weight and BMI did not differ at baseline.

### 3.1. Body Composition Outcome

The statistical analysis for body weight was executed following the intention-to-treat (ITT) principle, encompassing the total randomized sample of 60 participants. After adjusting for baseline weight as a covariate, the analysis of covariance (ANCOVA) revealed a statistically significant difference in mean body weight between the groups at the end of the 12-week intervention (F (3, 55) = 9.444, *p* < 0.001, ηp^2^ = 0.340). The calculated partial eta squared (ηp^2^ = 0.340) indicates a large effect size, suggesting that the experimental interventions exerted a robust impact on weight reduction.

Subsequent pairwise comparisons using the Bonferroni post hoc test provided detailed insights into the inter-group dynamics. Specifically, the TF group demonstrated a significant reduction in body weight compared to the P group (mean difference = −4.004 kg; *p* < 0.001; 95% CI −6.439, −1.569]). Similarly, the F group exhibited a statistically significant weight loss relative to the P group (mean difference = −3.933 kg; *p* < 0.001; 95% CI [−6.371, −1.494]). Furthermore, the TP group also showed a significant decrease in weight compared to the P group (mean difference = −3.617 kg; *p* = 0.001; 95% CI [−6.053, −1.182]).

The statistical analysis of the BMI was conducted using the intention-to-treat approach through an analysis of covariance model. After adjusting for baseline BMI values as a covariate, the results demonstrated a statistically significant difference between the groups at the conclusion of the 12-week intervention (F (3, 55) = 3.519, *p* = 0.021, ηp^2^ = 0.161). The observed partial eta squared (ηp^2^ = 0.161) indicates a large effect size, underscoring the substantial impact of the experimental protocols on BMI modulation within the study population.

Further examination via Bonferroni post hoc pairwise comparisons revealed that the TF group was the only group to achieve a statistically significant reduction in BMI compared to the P group (mean difference = −1.966 kg/m^2^; *p* = 0.024; 95% CI [−3.760, −0.172]). In contrast, the TP group did not reach the threshold for statistical significance relative to the P (mean difference = −1.623 kg/m^2^; *p* = 0.083; 95% CI [−3.372, 0.125]), nor did the F group (mean difference = −1.479 kg/m^2^; *p* = 0.156; 95% CI [−3.248, 0.290]). Notably, no statistically significant differences were observed among the three active intervention groups themselves (*p* = 1.000).

### 3.2. Biochemical and Inflammatory Outcomes

The statistical evaluation of asprosin concentrations was performed using the ITT approach through an analysis of covariance framework. After adjusting for baseline asprosin values as a covariate, the model revealed a highly significant main effect of the intervention type on asprosin levels (F (3, 55) = 36.047; *p* < 0.001). The observed partial eta squared (ηp^2^ = 0.663), indicating that 66.3% of the variance in asprosin reduction is directly attributable to the experimental interventions.

Subsequent pairwise comparisons using the Bonferroni post hoc test provided further insights into the comparative efficacy of the protocols. The TF group exhibited the most substantial and statistically significant decline in asprosin levels compared to the P group (mean difference = −0.567 ng/mL; *p* < 0.001; 95% CI [−0.728, −0.406]). Furthermore, the TP group also demonstrated a significant reduction in this adipokine relative to the P group (mean difference = −0.449 ng/mL; *p* < 0.001; 95% CI [−0.611, −0.288]). Notably, unlike previous parameters, the F group also achieved a statistically significant decrease in asprosin compared to the P (mean difference = −0.239 ng/mL; *p* = 0.001; 95% CI [−0.400, −0.079]).

The statistical analysis of MCP-1 levels was performed in accordance with the intention-to-treat principle using an ANCOVA. After adjusting for baseline MCP-1 values as a covariate, a highly significant difference was observed among the study groups at the conclusion of the intervention (F (3, 55) = 29.570, *p* < 0.001). The partial eta squared (ηp^2^ = 0.617), indicating that the experimental interventions accounted for 61.7% of the total variance in MCP-1 reductions.

Subsequent pairwise comparisons using the Bonferroni post hoc test provided a detailed characterization of the anti-inflammatory efficacy across the groups. The TF group exhibited the most substantial decrease in MCP-1 levels relative to the P group (mean difference = −83.053 pg/mL; *p* < 0.001; 95% CI [−108.691, −57.415]). Furthermore, significant reductions were recorded in the TP group (mean difference = −64.650 pg/mL; *p* < 0.001; 95% CI [−90.180, −39.119]) and the F group (mean difference = −57.030 pg/mL; *p* < 0.001; 95% CI [−82.590, −31.470]) when compared to the P group.

### 3.3. Adipokines and Leptin Outcomes

Statistical analysis of adiponectin levels, utilizing the ANCOVA model based on the ITT principle, revealed a significant main effect for the intervention type (F (3, 55) = 13.539; *p* < 0.001). According to the partial eta squared (ηp^2^ = 0.425), the experimental interventions accounted for 42.5% of the total variance in post-test adiponectin changes.

Bonferroni-corrected pairwise comparisons further elucidated the efficacy of the protocols; specifically, the TF group demonstrated a significant increase in adiponectin levels compared to the P (mean difference = 4.385 ng/mL; *p* < 0.001; 95% CI [2.078, 6.691]). Similarly, the TP group showed a significant elevation relative to the P (mean difference = 4.220 ng/mL; *p* < 0.001; 95% CI [1.924, 6.516]). Notably, the F intervention did not produce a statistically significant change compared to the P group (mean difference = 1.217 ng/mL; *p* = 0.912), despite the observed numerical difference.

The statistical analysis of leptin concentrations was conducted using the ANCOVA framework, following the intention-to-treat principle. After adjusting for baseline leptin levels as a covariate, the model demonstrated a statistically significant difference between the groups at the conclusion of the 12-week intervention (F (3, 55) = 2.925; *p* = 0.042). The reported partial eta squared (ηp^2^ = 0.138) reflects a medium effect size, suggesting that the experimental protocols exerted a moderate impact on the modulation of leptin levels within the studied population.

Further examination via Bonferroni post hoc pairwise comparisons revealed specific inter-group disparities. Notably, the TF group was the sole intervention to achieve a statistically significant reduction in leptin levels compared to the P group (mean difference = −1.604 ng/mL; *p* = 0.049; 95% CI [−3.202, −0.005]). In contrast, while the TP group (mean difference = −0.972 ng/mL; *p* = 0.603; 95% CI [−2.565, 0.621]) and the F group (mean difference = −1.375 ng/mL; *p* = 0.134; 95% CI [−2.977, 0.226]) exhibited downward trends in mean values, these changes did not reach statistical significance relative to the P group. Furthermore, no significant differences were observed among the three active intervention groups (*p* = 1.000).

### 3.4. Lipid Profile Outcomes

The statistical analysis of LDL-C concentrations was conducted using the ITT approach through an ANCOVA model. After adjusting for baseline LDL-C values as a covariate, the results demonstrated a highly potent and significant main effect of the intervention type on post-test concentrations (F (3, 55) = 183.271; *p* < 0.001). The calculated partial eta squared (ηp^2^ = 0.909) reflects an exceptionally large effect size, indicating that 90.9% of the total variance in LDL-C reduction is directly attributable to the experimental protocols.

Detailed pairwise comparisons using the Bonferroni post hoc test revealed profound inter-group disparities. The TF group exhibited a greater reduction compared to other groups and statistically significant decrease in LDL-C levels compared to the P group (mean difference = −18.745 mg/dL; *p* < 0.001; 95% CI [−21.326, −16.164]). Similarly, the TP-only group achieved a substantial and statistically significant reduction relative to the P group (mean difference = −18.649 mg/dL; *p* < 0.001; 95% CI [−21.286, −16.013]). Furthermore, the F group also recorded a significant improvement compared to the P group, albeit with a smaller magnitude of change (mean difference = −6.453 mg/dL; *p* < 0.001; 95% CI [−8.947, −3.959]).

The statistical analysis of HDL-C levels was conducted using the ITT principle within an ANCOVA framework. After adjusting for baseline HDL-C values as a covariate, the model revealed a highly significant main effect of the intervention type on concentrations (F (3, 55) = 44.555; *p* < 0.001). The observed partial eta squared (ηp^2^ = 0.708), suggesting that 70.8% of the total variance in HDL-C elevation is directly attributable to the experimental protocols implemented.

Further examination through Bonferroni post hoc pairwise comparisons provided a detailed characterization of group-specific outcomes. The TF group demonstrated the most substantial and statistically significant increase in HDL-C levels compared to the P group (mean difference = 7.961 mg/dL; *p* < 0.001; 95% CI [5.732, 10.191]). Similarly, the TP group exhibited a highly significant elevation relative to the P group (mean difference = 7.130 mg/dL; *p* < 0.001; 95% CI [5.068, 9.191]). In marked contrast, the F group did not reach statistical significance when compared to the P group (mean difference = 1.850 mg/dL; *p* = 0.062; 95% CI [−0.056, 3.756]).

The statistical analysis of TG levels was performed using the ITT principle within an ANCOVA framework. After adjusting for baseline TG values as a primary covariate, the results demonstrated that the type of intervention exerted a highly significant effect on triglyceride concentrations (F (3, 55) = 197.612; *p* < 0.001). The reported partial eta squared (ηp^2^ = 0.915) indicates a large effect size.

Further granular analysis via the Bonferroni post hoc test for pairwise comparisons revealed significant metabolic improvements across all active groups. The TF group showed the most substantial and statistically significant reduction in TG levels compared to the P group (mean difference = −30.569 mg/dL; *p* < 0.001; 95% CI [−34.808, −26.331]). Similarly, the TP group achieved a significant decline relative to the P group (mean difference = −27.251 mg/dL; *p* < 0.001; 95% CI [−31.549, −22.953]). Furthermore, the F group also exhibited a statistically significant improvement in TG levels compared to the P group (mean difference = −5.331 mg/dL; *p* = 0.009; 95% CI [−9.711, −0.950]).

The statistical evaluation of TC levels was conducted using the ITT approach through an ANCOVA model. After adjusting for baseline TC concentrations as a covariate, the analysis revealed a highly significant main effect of the intervention type on TC (F (3, 55) = 246.943; *p* < 0.001). The observed partial eta squared (ηp^2^ = 0.931) represents an exceptionally robust effect size, indicating that 85.9% of the total variance in TC reduction is directly attributable to the experimental protocols. This high degree of variance explanation underscores the clinical significance of the implemented interventions in lipid profile management.

Further granular insights provided by the Bonferroni post hoc pairwise comparisons confirmed significant improvements across all experimental cohorts. The TF group demonstrated the most substantial and statistically significant reduction in TC levels compared to the P group (mean difference = −25.746 mg/dL; *p* < 0.001; 95% CI [−28.671, −22.822]). Similarly, the TP group achieved a profound decrease relative to the P group (mean difference = −23.801 mg/dL; *p* < 0.001; 95% CI [−26.683, −20.920]). Additionally, the F group also showed a statistically significant improvement compared to the P group (mean difference = −8.605 mg/dL; *p* < 0.001; 95% CI [−11.037, −6.173]) (Table 4).

The Pearson correlation analysis revealed significant relationships between the studied markers (Table 5). Table 5 summarizes the interrelationships between the differential changes (Delta) of the investigated variables using Pearson’s correlation coefficient. The findings revealed robust and statistically significant associations between alterations in serum asprosin levels and the participants’ metabolic profiles. Specifically, changes in asprosin demonstrated strong positive correlations with fluctuations in LDL-C (r = 0.680, *p* < 0.01), TC (r = 0.838, *p* < 0.01), TG (r = 0.721, *p* < 0.01), and the inflammatory marker MCP-1 (r = 0.548, *p* < 0.01). Conversely, significant inverse correlations were observed between asprosin and changes in HDL-C (r = −0.770, *p* < 0.01) and Adiponectin (r = −0.404, *p* < 0.01).

Notably, within this cross-sectional analysis, no statistically significant association was detected between the changes in asprosin and Leptin levels (r = 0.083, *p* > 0.05). Furthermore, the most potent inverse relationship across the entire dataset was recorded between the changes in TG and HDL-C (r = −0.879, *p* < 0.01). To maintain the stringency of the findings and mitigate the risk of Type I error arising from multiple comparisons, all significance levels were rigorously evaluated and confirmed through the Bonferroni correction method.

## 4. Discussion

The present study demonstrated that 12 weeks of interval-style resistance and aerobic training, combined with fisetin supplementation, led to significant improvements in anthropometric indices, lipid profiles, and adipocytokine levels in men with obesity. Our findings indicate that the combined protocol (TF group) exhibited the highest efficacy in reducing body weight, BMI, and fat mass, which was accompanied by a substantial improvement in LDL-C, triglycerides, and total cholesterol levels. These results are consistent with previous research confirming the synergistic role of physical activity and polyphenolic compounds in improving the metabolic status of obese individuals [19,20]. Furthermore, the synergy between exercise and fisetin was evident in modulating systemic inflammation through a significant reduction in MCP-1 and an increase in HDL-C, suggesting the high potential of this dual intervention in modifying immuno-metabolic responses.

In the analysis of adipocytokines, the reduction in asprosin concentration was a pivotal finding of this study, with the greatest decrease observed in the exercise plus fisetin group. Asprosin, as a glucogenic hormone, is directly linked to insulin resistance and visceral fat accumulation [12,21]. The reduction in its levels in the current study is likely due to the decrease in fat mass and improved insulin sensitivity following high-intensity interval training, alongside the senolytic properties of fisetin, which effectively inhibits the secretion of this protein from adipocytes [12]. Moreover, emerging evidence emphasizes that asprosin is not only a metabolic factor but also a central regulator of appetite and energy balance; its reduction indicates the high potential of the exercise-fisetin combination in modifying the metabolic programming of adipose tissue [22].

Additionally, the increase in adiponectin levels in the training groups signifies improved adipose tissue function and enhanced oxidative metabolism [23]. This increase was inversely correlated with the reduction in asprosin and the improvement of the lipid profile, which serves as a driver for glucose and fatty acid oxidation [24,25]. The probable mechanisms for these changes can be attributed to the activation of the SIRT1/AMPK pathway, leading to increased fatty acid oxidation and suppression of asprosin production [26,27]. Fisetin, specifically by inhibiting the NF-κB inflammatory pathway and reducing the nuclear translocation of the p65 subunit, leads to decreased expression of chemokines such as MCP-1 in adipocytes [28,29,30]. On the other hand, the senolytic effects of fisetin on adipose tissue can improve the secretory environment of the tissue by reducing senescent cells, thereby increasing adiponectin [31,32]. Furthermore, the improvement in the lipid profile may result from the upregulation of the cholesterol clearance pathway by membrane transporters, which are jointly influenced by exercise and polyphenols [33]. Finally, the strong positive correlation between asprosin changes and lipid indices in this study highlights the role of this adipocytokine as a key prognostic marker.

### Limitation

The present study, while providing novel insights into the efficacy of combined exercise and supplementation interventions, is not without limitations that necessitate cautious interpretation and influence the generalizability of the findings. The relatively modest sample size in each experimental cohort may have constrained the statistical power required to detect subtle physiological variances between the groups. Although the 12-week intervention duration is empirically sufficient to induce significant metabolic and structural adaptations, the long-term sustainability of these effects beyond this period remains to be elucidated. Despite the novel insights provided regarding the synergistic efficacy of concurrent training and Fisetin supplementation, the present study is not without its methodological constraints. A primary limitation lies in the estimation of aerobic exercise intensity via predictive HRmax equations rather than direct maximal exhaustion protocols. The absence of Gold Standard cardiopulmonary exercise testing (CPET) to objectively establish maximal physiological thresholds represents a surrogate approach that may influence the absolute precision of the prescribed training loads. One limitation of the present study is the lack of continuous, daily dietary monitoring throughout the 12-week intervention. Although participants were instructed to maintain habitual intake and food records showed no significant changes at the end of the study, the possibility of transient dietary variations cannot be entirely ruled out.

## 5. Conclusions

In summary, the present study provides preliminary evidence that combining interval resistance–aerobic training with fisetin supplementation modulates the adipokine profile in men with obesity, notably through the reduction in circulating asprosin and MCP-1 levels and the elevation of adiponectin. These changes were accompanied by favorable shifts in traditional cardiometabolic risk markers, suggesting a potential synergistic effect of this combined intervention. However, given the short-term nature of this trial and the absence of direct molecular assays, these findings should be viewed as exploratory. Further research involving larger, more diverse cohorts and longer follow-up periods is essential to validate these outcomes and to determine if such a strategy can be integrated into clinical frameworks for the non-pharmacological management of obesity and metabolic inflammation.

## Figures and Tables

**Table 1:** Mean (±SD) daily nutrient intake in study groups before and after the intervention.

Variable	P		F		TP		TF	
	Pre	Post	Pre	Post	Pre	Post	Pre	Post
Energy (kcal/day)	2240 ± 72	2243 ± 79	2265 ± 95	2258 ± 91	2238 ± 105	2235 ± 98	2252 ± 107	2245 ± 102
CHO (g/day)	278 ± 14.1	279 ± 13.9	275 ± 15.6	274 ± 14.7	281 ± 16.5	280 ± 15.4	283 ± 17.2	281 ± 16.0
Fat (g/day)	80.5 ± 9.8	80.2 ± 9.7	82.1 ± 10.1	81.8 ± 9.4	79.9 ± 10.6	79.7 ± 10.0	80.6 ± 10.3	80.3 ± 9.8
Protein (g/day)	102 ± 11.3	102.5 ± 11.7	101 ± 12.0	100.8 ± 11.5	103.1 ± 12.6	102.8 ± 12.4	103.3 ± 13.0	102.9 ± 12.7

Control-Placebo (P), fisetin (F), Training-Placebo (TP), or Training-Fisetin (TF).

**Table 2:** Reasons for participant attrition across study groups.

Group	Allocated (n)	Lost to Follow-Up (n)	Specific Reasons for Attrition
**TF**	15	4	2: Joint disorders; 1: New medication; 1: Personal reasons
**TP**	15	4	2: Low training attendance; 2: Unrelated injuries
**F**	15	4	2: Started caffeine supplements; 2: Non-compliance with pills
**P**	15	4	2: Relocation; 1: Started new exercise program; 1: Personal reasons

**Table 3:** Characteristics of experimental and placebo participants in men with obesity.

Groups	Age (Years)	Height (cm)	Weight (kg)	BMI (kg/m^2^)
TP	28.58 ± 3.82	180.33 ± 4.56	105.27 ± 5.94	32.34 ± 0.67
TF	26.64 ± 3.41	180.72 ± 5.99	105.18 ± 5.71	32.16 ± 0.65
F	28.79 ± 4.04	179.54 ± 4.50	104.03 ± 5.52	32.25 ± 0.44
P	26.35 ± 4.21	179.29 ± 6.60	104.88 ± 8.26	32.58 ± 0.77
*p*-**Value**	0.331	0.921	0.974	0.474

Control-Placebo (P), Fisetin (F), Training-Placebo (TP), or Training-Fisetin (TF).

**Table 4:** Changes in some inflammatory factors and insulin resistance index in the research groups.

Stages						
Variables and Groups	Pre-Test M ± SD ^a^	12th Week M ± SD ^a^	Percentage of Changes	*p*-Value ^b^	ANCOVA (*p*-Value)	Effect Size ηp^2^
**Weight (kg)**						
TP (n = 15)	105.27 ± 5.02	102.81 ± 2.10	−2.39	0.168	**0.001**	0.340
TF (n = 15)	105.18 ± 6.35	102.42 ± 3.02	−2.69	0.063		
F (n = 15)	104.03 ± 4.67	102.43 ± 2.16	−1.44	0.223		
P (n = 15)	104.88 ± 2.33	106.41 ± 2.33	1.43	0.422		
**BMI (kg/m^2^)**						
TP (n = 15)	32.34 ± 0.57	31.68 ± 1.76	−2.08	0.220	**0.021**	0.161
TF (n = 15)	32.16 ± 0.55	31.40 ± 1.30	−2.42	0.086		
F (n = 15)	32.25 ± 0.37	31.86 ± 1.42	−1.22	0.330		
P (n = 15)	32.58 ± 0.65	33.22 ± 2.24	1.92	0.291		
**Asprosin (ng/mL)**						
TP (n = 15)	1.41 ± 0.26	0.96 ± 0.10	−46.87	**0.001**	**0.001**	0.663
TF (n = 15)	1.35 ± 0.18	0.84 ± 0.12	−60.71	**0.001**		
F (n = 15)	1.34 ± 0.15	1.17 ± 0.13	−14.52	**0.004**		
P (n = 15)	1.36 ± 0.35	1.41 ± 0.24	3.54	0.649		
**MCP-1 (pg/mL)**						
TP (n = 15)	213.66 ± 28.22	154.68 ± 28.78	−38.13	**0.001**	**0.001**	0.617
TF (n = 15)	199.49 ± 28.98	136.17 ± 15.92	−46.50	**0.001**		
F (n = 15)	214.84 ± 23.42	162.31 ± 27.22	−32.36	**0.001**		
P (n = 15)	208.37 ± 32.26	219.29 ± 26.98	4.97	0.254		
**Adiponectin (ng/mL)**						
TP (n = 15)	9.23 ± 1.68	13.07 ± 2.61	29.38	**0.002**	**0.001**	0.425
TF (n = 15)	9.59 ± 2.74	13.26 ± 2.37	27.67	**0.001**		
F (n = 15)	9.05 ± 2.39	10.05 ± 1.98	10.03	0.196		
P (n = 15)	8.87 ± 2.62	8.83 ± 2.08	−0.45	0.956		
**Leptin (ng/mL)**						
TP (n = 15)	5.55 ± 1.29	4.91 ± 1.52	−13.03	0.290	**0.042**	0.138
TF (n = 15)	5.40 ± 1.44	4.27 ± 1.60	−26.46	0.069		
F (n = 15)	5.34 ± 1.88	4.49 ± 1.00	−18.93	0.158		
P (n = 15)	5.88 ± 1.31	5.91 ± 2.01	0.50	0.949		
**LDL-C (mg/dL)**						
TP (n = 15)	139.82 ± 2.23	123.20 ± 2.47	−13.49	**0.001**	**0.001**	0.909
TF (n = 15)	139.14 ± 3.08	122.50 ± 2.13	−13.58	**0.001**		
F (n = 15)	137.56 ± 4.58	133.41 ± 4.43	−3.11	**0.001**		
P (n = 15)	136.22 ± 3.78	138.69 ± 5.66	1.78	**0.001**		
**HDL-C (mg/dL)**						
TP (n = 15)	35.16 ± 1.93	42.46 ± 1.02	17.19	**0.001**	**0.001**	0.708
TF (n = 15)	34.06 ± 2.81	43.26 ± 0.80	21.26	**0.001**		
F (n = 15)	36.79 ± 1.13	37.24 ± 3.37	1.20	**0.001**		
P (n = 15)	37.84 ± 2.36	35.42 ± 0.83	−6.83	**0.001**		
**TG (mg/dL)**						
TP (n = 15)	271.58 ± 6.32	244.39 ± 8.35	−11.12	**0.001**	**0.001**	0.915
TF (n = 15)	269.92 ± 5.03	239.85 ± 3.92	−12.53	**0.001**		
F (n = 15)	272.83 ± 5.01	267.23 ± 5.57	−2.09	**0.001**		
P (n = 15)	269.10 ± 3.71	269.81 ± 3.25	0.26	0.079		
**TC (mg/dL)**						
TP (n = 15)	248.00 ± 1.86	226.80 ± 2.43	−9.34	**0.001**	**0.001**	0.931
TF (n = 15)	248.29 ± 2.15	225.10 ± 2.62	−10.30	**0.001**		
F (n = 15)	244.44 ± 4.63	239.09 ± 4.39	−2.23	**0.001**		
P (n = 15)	239.70 ± 4.45	243.81 ± 4.45	1.68	**0.001**		

**Note**: Bold values indicate statistical significance (*p* < 0.05). Control-Placebo (P), Fisetin (F), Training-Placebo (TP), or Training-Fisetin (TF) ^a^ Values are expressed as mean ± standard deviation. ^b^ Within-group *p*-value. Results are based on Intention-to-Treat (ITT) analysis (N = 60) with missing values handled via mean imputation. One-way ANCOVA was used to compare post-intervention means among groups, using baseline values as covariates. Descriptive data (Mean ± SD) are reported for participants who completed the study, while *p*-values and effect sizes are derived from the ITT analysis (N = 60).

**Table 5:** Pearson correlation matrix between inflammatory and metabolic indices.

Variables	Asprosin	MCP-1	Adiponectin	Leptin	HDL-C	LDL-C	TC	TG
Asprosin	1							
MCP-1	0.548 **	1						
Adiponectin	−0.404 **	−0.417 **	1					
Leptin	0.083	0.341	−0.128	1				
HDL-C	−0.770 **	−0.536 **	0.551 **	−0.311	1			
LDL-C	0.680 **	0.694 **	−0.580 **	0.270	−0.739 **	1		
TC	0.838 **	0.593 **	−0.495 **	0.210	−0.821 **	0.804 **	1	
TG	0.721 **	0.553 **	−0.572 **	0.238	−0.879 **	0.769 **	0.869 **	1

**Note**: Sample size, N = 60. Values represent Pearson correlation coefficients based on the intention-to-treat (ITT) principle, with missing data managed via group-based mean imputation. The correlations are calculated using delta (Delta) values (post-intervention change). ** Correlation is statistically significant at the *p* < 0.01 level (2-tailed). The level of statistical significance was rigorously adjusted using the Bonferroni correction.

## Data Availability

The data presented in this study are available on request from the corresponding author due to privacy restrictions.
